# Supplementary material for: Identification of Freezing-Responsive microRNAs and Their Targets in Chinese Jujube by Small RNA and Degradome Sequencing
Source: Int J Mol Sci. 2026 Sep 4;27(17):7912. doi: 10.3390/ijms27177912 (PMC13566890; doi:10.3390/ijms27177912)
Supplement: Supplementary file 1 [file ijms-27-07912-s001.zip › Supplementary Material S2/novel_miR_133.pdf]

Provisional ID	:	novel_miR_133
Score total	:	58
Score for star read(s)	:	3.9
Score for read counts	:	56.2
Score for mfe	:	0.1
Score for randfold	:	-2.2
Score for cons. seed	:	
Total read count	:	122
Mature read count	:	72
Loop read count	:	40
Star read count	:	10

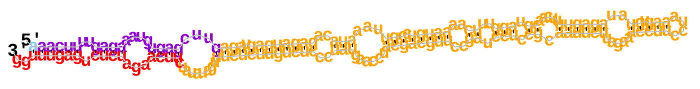

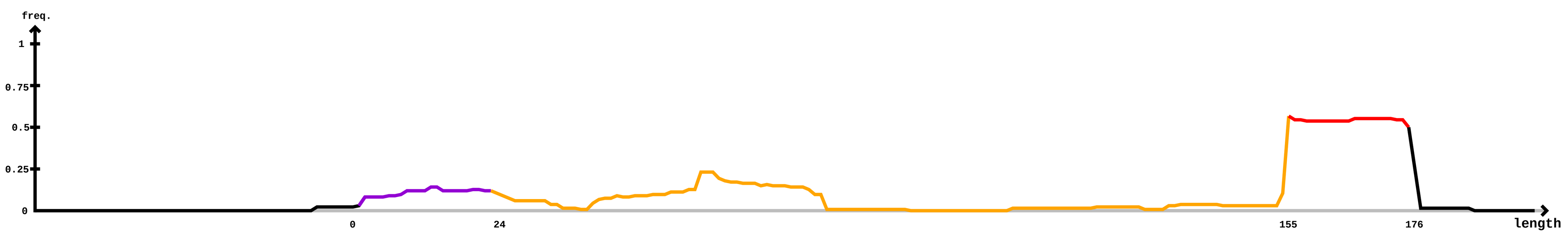

Star

Mature

Sequence		reads	mm	sample
5'- gcacgguuucuauugcugucucacucaaaucuaaauaucuuauauugggugcaaacuuuugagaaaugugagcuugagaugaguagagacauaaauugccuguaaaacuuuggaugcaacuuuugagauaugugaaauccuuccaaagauucuuaacgccuccuagccuugcagcauccaaguaucccucuguucucuuugauauaucuucaagaucucugaguuuggagcagccauuuagauuuagaua -3'	obs			
gcacgguuucuauugcugucucacucaaaucuaaauaucuuauauugggugcaaacuuuugagaaaugugagcuugagaugaguagagacauaaauugccuguaaaacuuuggaugcaacuuuugagauaugugaaauccuuccaaagauucuuaacgccuccuagccuugcagcauccaaguaucccucuguucucuuugauauaucuucaagaucucugaguuuggagcagccauuuagauuuagaua	exp			
..(((((.......))))).........((((((..((((.....(((.(((((((((..((((....((((...((((.((((((((..(((...(((.(((((..((..(((.((.....((((((..((.(((....)))))).....)))))).)).))).))..)))))))).....)))..))))))))))))).........))))...)))).))))))...))).)))...))))))))).		reads	mm	sample
ugggugcaaacuuuugGgaaa		2	1	JCK-2
ugggugcaaacuuuugGgaaa		1	1	JCK-3
aaacuuuugGgaaaugugagc		1	1	JCK-2
aacuuuugagaaaugugagcuug		7	0	LCK-3
uuugaAaaaugugagcuugag		1	1	LT-3
ugagaaaugugagcuugag		1	0	LT-3
gagaaaugugagcCugagaugagu		3	1	LT-1
aaugugagcuugagaugaguag		2	0	LT-2
aaugugagcuugagaugCguag		1	1	LT-2
gcuugagaugaguagaga		1	0	LT-1
agaugaguagagacauaaau		1	0	LT-3
uaaauugccuguaaaUcuuug		1	1	LT-2
uaaauugccuguaaaUcuuug		1	1	LT-3
uaaauugccuguaaaUcuuug		3	1	LT-1
aaauugccuguaaaCcuuugg		2	1	JCK-3
aaauugccuguaaaacuuuggaug		1	0	JCK-1
aauugcUuguaaaacuuugga		1	1	LT-1
uugccuguaaaacuuuggaugcaa		2	0	LT-1
ccuguaaaacuuuggaugcaacu		1	0	LT-1
Auaaaacuuuggaugcaacuuuu		1	1	LT-1
aaacuuuggaugcaacuuuugag		2	0	LT-2
cuuuggaugcaacuuuuCaga		1	1	LT-1
cuuuggaugcaacuuuugaga		1	0	JCK-3
uuggaugcaacuuuuCaga		1	1	JCK-2
uuggaugcaacuuuuCaga		1	1	JCK-1
uuggaugcaacuuuugagaua		11	0	JT-1
uuggaugcaacuuuugagaua		1	0	LT-3
uuuugagauaugugaaauccuucc		1	0	LCK-3
ccuagccuugcagcauccaagu		2	0	JCK-2
auccaaguaCcccucuguucu		1	1	JCK-3
cucuguucucuuugauauauc		1	0	JCK-3
cucuguucucuuugauauauc		2	0	JCK-1
cuguucucuuugauauaucuu		1	0	LT-1

```
                                                     Star                                                                                                                                                                  Mature
gcacgguuucuauugcugucucacucaaaucuaaauaucuuauauugggugcaaacuuuugagaaaugugagcuugagaugaguagagacauaaauugccuguaaaacuuuggaugcaacuuuugagauaugugaaauccuuccaaagauucuuaacgccuccuagccuugcagcauccaaguaucccucuguucucuuugauauaucuucaagaucucugaguuuggagcagccauuuagauuuagaua
..............................................................................................................................................................................................................ucuucaagaucucugaguu.........................	1	0	JCK-2
..............................................................................................................................................................................................................ucuucaagaucucugaguuug.......................	2	0	JCK-1
..............................................................................................................................................................................................................ucuucaagaucucugaguuug.......................	1	0	JCK-2
..............................................................................................................................................................................................................ucuucaagaucucugaguuug.......................	3	0	LT-2
..............................................................................................................................................................................................................ucuucaagaucucugaguuugg......................	1	0	JCK-2
..............................................................................................................................................................................................................ucuucaagaucucugaguuugg......................	2	0	JCK-3
...............................................................................................................................................................................................................cuucaagaucucugaguuugg......................	13	0	JCK-2
...............................................................................................................................................................................................................cuucaagaucucugaguuugg......................	3	0	LT-1
...............................................................................................................................................................................................................cuucaagaucucugaguuugg......................	12	0	LCK-3
...............................................................................................................................................................................................................cuucaCgaucucugaguuugg......................	1	1	LT-3
...............................................................................................................................................................................................................cuucaagaucucugaguuugg......................	9	0	JCK-3
...............................................................................................................................................................................................................cuucaagaucucugaguuugg......................	5	0	JCK-1
...............................................................................................................................................................................................................cuucaagaucucugaguuugg......................	5	0	LT-3
...............................................................................................................................................................................................................cuucaagaucucugaguuugg......................	14	0	LT-2
..........................................................................................................................................................................................................................cugaguuuggagcagccaCu............	2	1	LT-3
```
